# Cytokine preactivation and ADCC: a potent strategy for enhancing human NK cell effector functions against 3D tumor models

**DOI:** 10.3389/fimmu.2026.1797290

**Published:** 2026-05-08

**Authors:** Ainara Lopez-Pardo, Ainhoa Amarilla-Irusta, Ainhoa Iturbe-Larrondo, Itxaso San Juan, Víctor Sandá, Olatz Zenarruzabeitia, Francesca D Ciccarelli, Francisco Borrego, Laura Amo

**Affiliations:** 1 Immunopathology Group, Biobizkaia Health Research Institute, Barakaldo, Spain; 2 Department of Genetics, Physical Anthropology and Animal Physiology, Faculty of Science and Technology, University of the Basque Country, Leioa, Spain; 3 Centre for Cancer Evolution, Barts Cancer Institute, Queen Mary University London, London, United Kingdom; 4 Cancer Systems Biology Laboratory, The Francis Crick Institute, London, United Kingdom; 5 Universita’ degli Studi di Milano La Statale, Milan, Italy; 6 Ikerbasque, Basque Foundation for Science, Bilbao, Spain

**Keywords:** ADCC, cetuximab, colorectal cancer, cytokines, immunotherapy, lung cancer, memory-like, NK cells

## Abstract

The adoptive transfer of NK cells has shown clinical promise in hematologic malignancies, but its efficacy in solid tumors remains limited. Three-dimensional (3D) models reproduce tumor architecture and immunosuppressive microenvironments more accurately than conventional two-dimensional (2D) cultures. Here, we employed colorectal cancer (CRC) and lung cancer 3D tumor models to evaluate the anti-tumor activity of cytokine-induced memory-like (CIML) NK cells and to test whether cetuximab augments these responses through antibody-dependent cellular cytotoxicity (ADCC). Human NK cells were preactivated overnight with interleukin (IL)-12/15/18 (pre-CIML), then co-cultured with tumor spheroids and patient-derived organoids in the presence or absence of cetuximab. Pre-CIML NK cells showed significantly enhanced effector functions compared to control NK cells, including increased degranulation, higher IFN-γ and TNF-α production, and superior cytotoxicity against both spheroids and organoids. Additionally, pre-CIML NK cells that infiltrated tumor spheroids displayed a more uniform distribution within the tumor mass than control NK cells, which may contribute to their improved killing capacity. Cetuximab-mediated ADCC further enhanced NK cell activity against spheroid models, while in organoids, the enhancement was tumor- and context-dependent. Overall, these findings demonstrate that pre-CIML NK cells exhibit robust anti-tumor activity in clinically relevant 3D tumor models, and identify ADCC as an additional, but context-restricted, mechanism by which targeted antibodies such as cetuximab can further enhance NK cell functionality. These findings underscore the translational potential of NK cell-based immunotherapies that combine cytokine preactivation and ADCC induction to overcome current challenges in the treatment of solid tumors.

## Introduction

Natural killer (NK) cells are cytotoxic lymphocytes belonging to the innate lymphoid cell (ILC) family. In humans, NK cells typically comprise 5–15% of circulating lymphocytes in peripheral blood and are generally identified as CD3−CD14−CD19− lymphocytes that express CD56. A key distinguishing feature of NK cells is their ability to rapidly recognize and eliminate abnormal cells, such as virus-infected or tumor cells, without the need of prior sensitization, unlike T cells, which require antigen priming. NK cell activation and effector functions are tightly regulated by the complex balance of signals received from cell surface activating and inhibitory receptors (1, 2).

Among the activating receptors, CD16a (FcγRIIIa) recognizes the fragment crystallizable (Fc) portion of IgG antibodies. This recognition triggers NK cell activation and the subsequent elimination of the target cell in a process known as antibody-dependent cellular cytotoxicity (ADCC) (3). Once activated, NK cells rapidly release cytolytic granules containing perforin and granzymes to induce target cell death. This potent mechanism is exploited therapeutically, particularly through the use of monoclonal antibodies (mAbs) for targeted cancer therapy (4, 5). A clinically relevant example is cetuximab, an IgG1 mAb that targets the epidermal growth factor receptor (EGFR), which is widely utilized in the treatment of colorectal cancer (CRC) and head and neck squamous cell carcinoma (HNSCC) (6, 7).

Given the crucial role of NK cells in cancer immune surveillance and their inherent suitability for allogeneic “off-the-shelf” applications, without the risk of inducing graft-versus-host disease, NK cell-based therapies are currently under development and investigated in clinical trials (1, 8–10). Some of these therapeutic strategies aim to increase the efficacy of adoptively transferred NK cells. The approaches include several cytokine stimulation strategies (such as interleukin (IL)-2/IL-15 or feeder cells-based expansion), combination with mAbs that exploit ADCC as a mechanism of action or with immune checkpoint inhibitors, and/or antigen-specific NK cell engineering (1, 11–19). Among these, the adoptive transfer of cytokine-induced memory-like (CIML) NK cells has emerged as a promising immunotherapy for different malignancies (18, 20, 21). These cells are generated by briefly stimulating NK cells with a combination of IL-12, IL-15 and IL-18 for 16–18 hours, which gives rise to NK cells with enhanced effector functions. Following cytokine removal and a resting period, cells retain a heightened state of readiness, which is characterized by enhanced cytotoxicity, superior cytokine production, and prolonged persistence *in vivo* (22, 23). Although the classical definition of CIML NK cells requires a subsequent resting period, these preactivated cells are often directly infused into patients in clinical settings and are also referred to as CIML NK cells (i.e. NCT01898793, NCT02782546, NCT03068819, NCT04024761, and NCT04634435). Therefore, in this study, we used the term “pre-CIML” NK cells to refer to NK cells analyzed immediately following the 16–18 hours cytokine preactivation period, without the additional resting phase. Their application is already yielding favorable outcomes against hematological malignancies, demonstrating that the initial cytokine boost alone is a powerful clinical strategy (18, 21).

Although the adoptive transfer of NK cells has shown substantial efficacy in hematologic malignancies, preclinical and clinical data indicate that its effectiveness in patients with solid tumors is hindered by several factors, including the immunosuppressive tumor microenvironment (TME), tumor immune evasion mechanisms, poor NK cell infiltration into tumor sites, and the lack of predictive, physiologically relevant models for therapy development (24, 25). To better understand NK cell functionality in solid tumors and to design improved NK cell-based therapies, more accurate *in vitro* models that recapitulate tumor environment are needed. In this context, three-dimensional (3D) culture models, such as tumor spheroids and tumor organoids, have been developed. Tumor spheroids are widely used, scaffold-free 3D models formed by the aggregation of cancer cell lines or patient-derived cells. These structures self-organize into microtumor-like architectures that mimic tumor features such as hypoxia, cell-cell cohesion and interaction, and nutrient gradients (26, 27). Tumor organoids, by contrast, are more complex 3D models derived from patient tumor tissues that preserve key histopathological, genetic and phenotypic features of the original tumor and are able to retain tumor cell heterogeneity to a greater extent (28, 29).

Recent advances in NK cell-based immunotherapy have incorporated 3D tumor models to investigate NK cell activity in cervical, gastric cancer, CRC, and other solid tumors (30–33). However, the evaluation of CIML NK cells, alone or in combination with mAbs such as cetuximab, in the context of 3D tumor models remains very limited. To address this gap, we investigated the anti-tumor responses of these NK cells in colorectal and lung cancer spheroids, as well as in patient-derived CRC organoids. We assessed multiple NK cell functions, including degranulation, interferon gamma (IFN-γ) and tumor necrosis factor alpha (TNF-α) production, anti-tumor cytotoxicity, and tumor infiltration. Our findings demonstrate that pre-CIML NK cells display enhanced effector functions against 3D tumor models. Moreover, ADCC-triggering mAbs, such as cetuximab, further potentiate NK cell activity in these systems. This approach provides a more comprehensive understanding of NK cell behavior in solid tumors and highlights the translational relevance of cytokine preactivation and ADCC-enhancing strategies to improve NK cell-based immunotherapies against solid tumors.

## Materials and methods

### A549 and HCT-116 cell cultures

The human lung adenocarcinoma cell line A549 was cultured in Dulbecco´s Modified Eagle Medium (DMEM, Corning) supplemented with 5% heat-inactivated Fetal Bovine Serum (FBS, Gibco), 1% MEM Non-Essential Amino Acids (NEAA, Gibco) and 1% Penicillin/Streptomycin (P/S, Gibco). This culture medium is hereinafter referred to as complete DMEM or cDMEM. Wild-type HCT-116 and ZsGreen-transfected HCT-116 (HCT-116-Green) CRC cell lines were kindly provided by Dr. Julián Pardo (IIS Aragón) and maintained under the same conditions. All cell lines were cultured at 37 °C in a humidified atmosphere containing 5% CO_2_.

### NK cell isolation

Buffy coats from healthy donors were provided by the Basque Biobank (https://www.biobancovasco.bioef.eus/). All donors provided written and signed informed consent in accordance with the Declaration of Helsinki. The study protocol was approved by the Basque Ethics Committee for Research with Medicines (CEIm-E) (PI+CES-BIOEF 2020-10).

Peripheral blood mononuclear cells (PBMCs) were isolated from buffy coats via Ficoll^®^ Paque Plus (GE Healthcare) density gradient centrifugation, as previously described (34). Freshly isolated PBMCs were either cryopreserved for later use (34) or immediately processed for NK cell isolation using the Human NK Cell Isolation Kit (Miltenyi Biotec). All the experiments were performed with purified NK cells (typically > 80% CD3−CD14−CD19−CD56+ cells).

Purified NK cells were plated at 2×10^6^ cells/mL in 6-well plates in Roswell Park Memorial Institute (RPMI) 1640 medium with L-glutamine (Corning), 10% heat-inactivated FBS, 1% NEAA, 1% sodium pyruvate (Gibco) and 1% P/S (hereinafter, complete RPMI or cRPMI). Cells were cultured overnight (16–18 hours) at 37 °C with 10 ng/mL recombinant human (rh) IL-12 (Miltenyi Biotec), 50 ng/mL rhIL-15 (Miltenyi Biotec) and 50 ng/mL rhIL-18 (MBL International Corporation), or in medium alone to obtain pre-CIML NK cells or control NK cells, respectively. After overnight incubation, cells were washed three times with cRPMI and subsequently processed according to the requirements of each co-culture assay (online **Supplementary Figure 1**).

### Generation of spheroids

Spheroid formation and characterization were performed as reported in (35). Briefly, between 1,000 and 10,000 A549, HCT-116 or HCT-116-Green cells were seeded in 96-well round-bottom ultra-low attachment plates (Corning) in 100 µL cDMEM per well. Cell line-specific densities per well were optimized for efficient spheroid formation based on the experimental assay. Plates were centrifuged at 300 g for 1 minute to promote cell aggregation. A549 and HCT-116 cell lines generated a mature spheroid per well in approximately 48 hours, as confirmed by microscopy. Detailed protocols for cell seeding density optimization, spheroid viability, growth rate analyses, and co-culture techniques, are described in (35).

### Organoid establishment and culture

CRC organoids used in this study were established, sequenced and cultured as previously described (36). Briefly, organoids were established from fresh tumor tissues obtained from patients who provided written informed consent, under ethical approvals REC 12-EE-0493 and 18-EE-0025 and subsequently stored under HTA License 12121. Genomic DNA from the organoids was sequenced by the Advanced Sequencing Facility at the Francis Crick Institute.

Organoid medium was prepared using Ad-DF+++ as the basal medium (consisting of Advanced DMEM/F-12 (Gibco) supplemented with 1% GlutaMAX (Gibco), 1% HEPES (Gibco), and 1% P/S), and adding the following components to the basal medium: 100 ng/mL rh Noggin (Prepotech), 40% 3dGRO R-Spondin-1 Conditioned Media Supplement (Merck), B-27™ supplement (Gibco), 50 ng/mL rh EGF (Stem Cell Technologies), 10 nM prostaglandin E2 (Tocris Biosciences), 1.25 mM N-Acetyl-L-cysteine, 10 mM nicotinamide, 500 nM TGF-β/ALK5 inhibitor A83-01, 10 μM p38 MAPK inhibitor SB202190, and 10 nM [Leu15]-Gastrin I human (all from Sigma-Aldrich).

Organoids were embedded in a mixture of 70% Geltrex (Geltrex™ LDEV-free reduced growth factor basement membrane extract, Gibco) and 30% of organoid medium, seeded in small droplets in 6-well plates, and left for 30 minutes at 37 °C and 5% CO_2_. After solidification of the droplets, organoid medium supplemented with 10 μM Rock inhibitor Y-27632 dihydrochloride (Sigma-Aldrich) was added. Organoids were maintained at 37 °C and 5% CO_2_ and passaged every 2 weeks by replacing culture medium with cold Ad-DF+++ to detach the geltrex droplets. The suspension was collected and washed by centrifugation at 300 g for 5 minutes at 4°C. The supernatant was then removed, and organoids were incubated with TrypLE Express (Gibco) for 5–10 minutes at 37 °C to facilitate their dissociation. The reaction was stopped by adding 5% FBS and samples were then washed with Ad-DF+++. Next, after a mechanical dissociation by pipetting, organoids were washed and resuspended in fresh 70% Geltrex/30% organoid medium. The suspension was replated in 6-well plates, splitting at ratios between 1:3 and 1:6 depending on growth rate. When required, organoids were cryopreserved in Recovery™ Cell Culture Freezing Medium (Thermo Fisher Scientific) until needed for further use.

### NK cell functional assay

To assess NK cell degranulation and cytokine production in response to 3D tumor models, NK cells were co-cultured with either tumor spheroids or organoids (online **Supplementary Figure 1**). Equal numbers of live pre-CIML or control NK cells were resuspended in cRPMI and added to spheroid cultures in 96-well U-bottom ultra-low attachment plates at a final volume of 200 µL per well. Effector to target (E:T) ratios were set at 1:1, based on the initial tumor cell seeding density: 10^4^ live cells/well for A549 and 5×10³ cells/well for HCT-116 wild-type cells.

For NK cell co-culture with tumor organoids, organoids were first collected and a fraction of them was dissociated to single cells and counted in order to estimate tumor cell content per organoid. Whole organoids, estimated to contain 2×10^5^ cells, were resuspended in cRPMI and added per well in a 24-well plate pre-treated with Anti-Adherent Rinsing Solution (STEMCELL Technologies). Equal number of live pre-CIML or control NK cells were then added at 2:1 E:T ratio, in a final volume of 500 µL cRPMI per well.

ADCC was induced in NK cell-spheroid and NK cell-organoid co-cultures by adding 1 µg/mL cetuximab (Erbitux 100 mg/20 mL, Merck) to the corresponding wells. Anti-CD107a-FITC (clone REA792, Miltenyi Biotec) was added at the start of co-cultures (0.4 μL/well for spheroids, 2 μL/well for organoids). After 1 hour, 1 μL/mL Golgi-Plug and 0.66 μL/mL Golgi-Stop (both from BD Biosciences) were added. The co-culture was maintained for an additional 16–18 hours (spheroids) or 4 hours (organoids), based on prior optimization and reported protocols (35, 37).

At the end of the culture, all samples were washed in phosphate buffered saline (PBS) by centrifugation at 300 g for 5 minutes. Spheroid co-cultures and their NK cell-only controls were digested with Trypsin-EDTA (Gibco) for 5–15 minutes until complete dissociation and filtered through 35 µm nylon mesh cell strainer caps. Organoid co-cultures and their NK cell-only controls were directly filtered without enzymatic dissociation (37). All samples were washed with PBS and stained with LIVE/DEAD Fixable Near-IR dye (Thermo Fisher Scientific) for 30 minutes at 4 °C. After two washes with 2.5% Bovine Serum Albumin (BSA)-PBS, cells were stained with anti-CD3-BV510 (clone UCHT1), anti-CD14-BV510 (clone MφP9), anti-CD19-BV510 (clone SJ25C1) and anti-CD7-PE (clone M-T701), all of them from BD Biosciences. Although CD56 is the canonical marker for NK cells and was initially tested, its expression was lost following the spheroid dissociation protocol, which includes trypsinization. Therefore, NK cells in these co-culture assays were defined as CD3⁻CD14⁻CD19⁻CD7⁺, as CD7 is constitutively expressed by the NK cell lineage and allows for their reliable identification, particularly when using purified NK cells (38). Next, for intracellular staining, samples were washed twice in 2.5% BSA-PBS, fixed and stained with anti-IFN-γ-PE-Cy7 (clone B27, BD Biosciences) and anti-TNF-α-APC (clone mAb11, Biolegend) using the Cytofix/Cytoperm kit (BD Biosciences), according to the manufacturer’s instructions. Finally, samples were washed, resuspended in 250 µL PBS, and acquired on a BD LSRFortessa X-20 flow cytometer. Flow cytometry data was analyzed using FlowJo 10.8.1.

### Spheroid killing assay

To assess NK cell-mediated cytotoxicity against tumor spheroids, equal number of live control or pre-CIML NK cells were resuspended in cRPMI and added to pre-formed spheroids in 96-well U-bottom ultra-low attachment plates at a final volume of 200 µL per well (online **Supplementary Figure 1**). For this assay, E:T ratios were set at 5:1, based on initial tumor cell seeding densities: 5x10^3^ live cells/well for A549 and 1.5×10³ live cells/well for HCT-116 parental cells. At the start of the co-culture, CellEvent™ Caspase-3/7 Green probe (Thermo Fisher Scientific) was added at 1:100 dilution, and 1 µg/mL cetuximab was added to the corresponding conditions. After 24 hours, transmitted light and fluorescence images were acquired using a Primovert Inverted Microscope (Zeiss) and analyzed using FIJI (ImageJ) software. Cytotoxicity was quantified based on the green fluorescence intensity in a selected region of interest (ROI) strictly within the spheroid area. Wells containing tumor spheroids alone were used to determine background fluorescence.

### NK cell infiltration assay

Pre-CIML or control NK cells were fluorescently labelled with eFluor™ 670 Cell Proliferation Dye (eBioscience) according to the manufacturer’s instructions. After washing with PBS, equal numbers of live cells were co-cultured with HCT-116-Green spheroids, in 96-well U-bottom ultra-low attachment plates, at a final volume of 200 µL per well (online **Supplementary Figure 1**). The E:T ratio was set at 3:1, based on an initial tumor cell seeding density of 10^3^ live cells/well. Cetuximab was added at 1 µg/mL to the designated conditions. For each NK cell donor and condition, three spheroids were prepared.

After 20 hours of co-culture, spheroids were transferred to chamber slides for confocal imaging. Images were acquired using a Zeiss LSM 880 Airyscan confocal microscope equipped with a Plan-Apochromat 10x/0.45 M27 objective. Z-stack images were captured in three channels: GFP, Cy5 and transmitted light with a transmitted photomultiplier tube (T-PMT), typically spanning 120–180 µm in depth with 9–10 µm intervals, to ensure full coverage of infiltrating NK cells. Maximum intensity projections of the Z-stacks were generated using FIJI software in all three channels. NK cell infiltration was assessed by quantifying the Cy5-positive area within the spheroid perimeter defined using the T-PMT projection. A qualitative scoring system was also used: randomized images were independently evaluated by three blinded observers who assigned scores from 1 to 3 based on NK cell distribution (1: infiltration but confined to a single region; 2: infiltration into multiple regions with a non-uniform scattered distribution; 3: uniform distribution throughout the spheroid). From the T-PMT projections, images were randomized and scored based on the degree of spheroid disruption (1: spheroid with preserved integrity; 2: partial damage; 3: loss of structural integrity), and spheroid circularity and area were quantified. From the GFP channel, GFP-positive area, circularity and total integrated intensity were quantified.

### Organoid killing assays

To assess the sensitivity of tumor organoids to NK cell-mediated killing, whole organoids, estimated to contain 2×10^4^ cells, were resuspended in 100 µL cRPMI and added per well in a 96-well U-bottom ultra-low attachment plate. Equal numbers of live control or pre-CIML NK cells were added in cRPMI to achieve a 5:1 E:T ratio, in a final volume of 200 µL per well (online **Supplementary Figure 1**). When indicated, 1 µg/mL of cetuximab was added to the co-cultures. 2 µL of CellEvent™ Caspase-3/7 Green probe was added to each well to monitor cell death.

Real-time imaging of co-cultures was performed using the xCELLigence RTCA eSight system (Agilent). GFP and brightfield images were captured every 15 minutes during the first hour, and then every hour for up to 24 hours. Images and videos were processed with RTCA eSight Software (Agilent) to quantify Green Total Integrated Intensity (GRI × µm²/image) and Green Total Area (µm²/image), both indicative of cell death in the co-cultures. Exported data were analyzed to evaluate the kinetics of tumor cell death over time. Quantitative indicators of NK cell-mediated killing included the area under the curve (AUC) and the initial slope of the increase in green area and in green fluorescence intensity. In addition, tumor area and fluorescence intensity were specifically assessed at the 24-hour time point.

### Statistical analysis

GraphPad Prism 9.5.1 and SPICE v6.1 (Vaccine Research Center, NIAID) softwares were used for graphical representation and statistical analysis. Data distribution was assessed using the Kolmogorov-Smirnov test. Depending on the distribution and experimental design, paired t-test, unpaired t-test or two-way Anova test were employed for parametric distributions, and Wilcoxon matched-pairs signed-rank test for non-parametric distributions, as specified in each figure legend. Differences between pie charts were determined with the non-parametric permutation test with 100,000 iterations. Statistical significance was defined as p<0.05; significance values are indicated as *(p<0.05), **(p<0.01), ***(p<0.001) and ****(p<0.0001). Group sizes and definition of error bars are indicated in figure legends.

## Results

### Pre-CIML NK cells degranulate and produce anti-tumor cytokines in response to tumor spheroids, and this is enhanced by cetuximab

**Figure 1:**
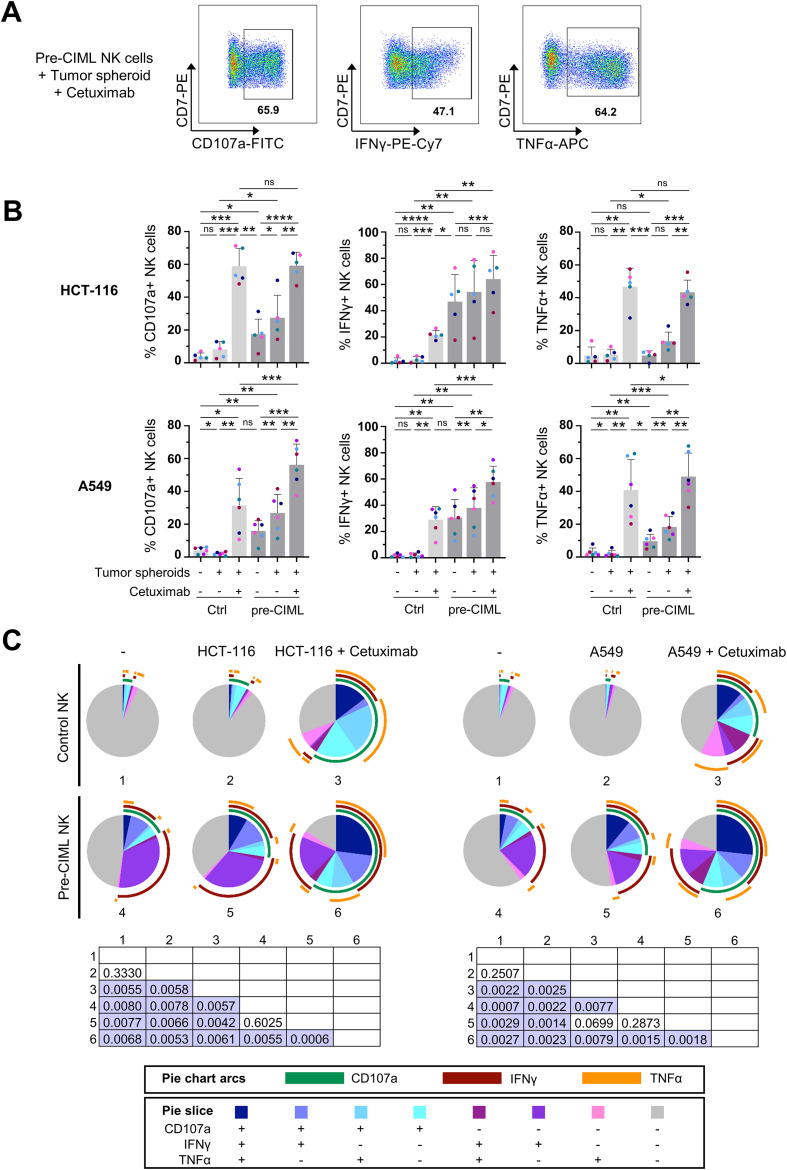
NK cell degranulation and cytokine production in response to tumor spheroids. Control or pre-CIML NK cells were co-cultured with tumor spheroids (1:1 E:T ratio) in the presence or absence of 1 µg/mL cetuximab for 16–18 hours. **(A)** Representative pseudocolor plots showing the percentage of pre-CIML NK cells expressing CD107a, IFN-γ or TNF-α after co-culture with A549 spheroids in the presence of cetuximab. **(B)** Bar graphs showing the percentage of NK cells expressing CD107a (left), IFN-γ (center) and TNF-α (right) after co-culture with HCT-116 (top panel), or A549 (bottom panel) spheroids. Bars show the mean ± SD, with individual color-coded donors (n=5 and n=6). Statistical analysis was performed using paired t-tests and significance is indicated as *(p<0.05), **(p<0.01), ***(p<0.001) and ****(p<0.0001). ns: non-significant. **(C)** Pie charts representing the proportions of NK cells expressing CD107a, IFN-γ and/or TNF-α in the indicated culture conditions. The slices indicate cells expressing different combinations of markers and the arcs denote which individual marker they express. Differences between conditions were assessed with a non-parametric permutation test (n=5 for HCT-116; n=6 for A549) and p-values are shown in the matrix. Significant differences are highlighted in purple.

CIML NK cells, characterized by enhanced activation and increased production of anti-tumor cytokines in response to tumor cells, have demonstrated considerable potential for the treatment of hematological malignancies (18, 20, 21). To assess their functionality against 3D culture systems, we first evaluated NK cell degranulation (CD107a) by flow cytometry after co-culture with tumor spheroids derived from lung (A549) and colorectal (HCT-116) cancer cell lines (**Figure 1A**; online **Supplementary Figure 2**). Control NK cells, cultured without prior stimulation, displayed very low frequencies of CD107a+ cells, which remained largely unchanged upon exposure to tumor spheroids (**Figure 1B**). In contrast, NK cells cultured overnight with IL-12/15/18 (termed as “pre-CIML NK cells”) showed a significantly higher proportion of CD107a expressing cells even in the absence of tumor targets, and this response was further significantly increased upon co-culture with both A549 and HCT-116 spheroids. We next analyzed IFN-γ and TNF-α production as key indicators of NK cell activity and function. Consistent with the degranulation data, control NK cells showed minimal cytokine production that did not appreciably increase upon exposure to tumor spheroids (**Figure 1B**). Although there was no (HCT-116) or small (A549) increase observed in the proportion of TNF-α+ cells for pre-CIML NK cells, the frequency of IFN-γ+ cells was markedly elevated, and these frequencies were increased upon co-culture with tumor spheroids (**Figure 1B**). These findings confirm that IL-12/15/18 preactivation primes NK cells for enhanced basal activity and potentiates their responsiveness to tumor-derived signals in a 3D context.

Given that both A549 and HCT-116 spheroids express EGFR (online **Supplementary Figures 3A, B**), we next explored whether ADCC induction with the addition of cetuximab (anti-EGFR mAb) could further enhance NK cell responses. In control NK cells, cetuximab significantly increased the frequencies of CD107a+, IFN-γ+ and TNF-α+ cells when co-cultured with either A549 or HCT-116 spheroids (**Figure 1B**). Remarkably, in some conditions the frequencies of functional control NK cells during ADCC surpassed those of pre-CIML NK cells cultured alone or even with tumor spheroids, particularly for CD107a and TNF-α. In pre-CIML NK cells, cetuximab addition further enhanced the frequencies of CD107a+, IFN-γ+ and TNF-α+ cells. In line with previously reported data (22, 23), pre-CIML NK cells displayed high baseline frequencies of IFN-γ+ cells even in the absence of stimulation. Thus, although cetuximab increased IFN-γ+ frequencies in pre-CIML NK cells, the relative effect was more pronounced in control NK cells. Notably, the presence of cetuximab in control NK cells co-cultured with A549 spheroids induced IFN-γ+ frequencies comparable to those of unstimulated pre-CIML NK cells. However, on a per-cell basis (median fluorescence intensity or MFI), IFN-γ production was higher in unstimulated pre-CIML NK cells compared to control cells (online **Supplementary Figure 4A**). Overall, cetuximab boosted NK degranulation and production of TNF-α, with a lower but still significant impact on IFN-γ production.

To obtain an integrated view of NK cell function, we analyzed NK cell polyfunctionality, defined as the capacity of individual cells to degranulate and/or produce multiple cytokines simultaneously (39). Most control NK cells were non-functional (CD107a−IFN-γ−TNF-α−), and exposure to tumor spheroids alone did not significantly improve their polyfunctionality, showing just a slight increase, mainly in response to HCT-116 spheroids (**Figure 1C**). Cetuximab addition to control NK cell co-cultures significantly increased the frequency of polyfunctional cells as observed by the emergence of populations expressing two or three functional markers (**Figure 1C**). Pre-CIML NK cells, with and without tumor spheroids, were consistently more functional than their respective control NK cells, with the highest levels of polyfunctionality observed in the presence of cetuximab (**Figure 1C**). Interestingly, in control NK cells treated with cetuximab, the non-functional population was smaller than in pre-CIML NK cells in the absence of cetuximab, with a marked increase in triple-positive NK cells, supporting the strong anti-tumor enhancing potential of cetuximab-induced ADCC. Although for A549 spheroids, control NK cells plus cetuximab did not show a statistically significant polyfunctional improvement compared to pre-CIML NK cells cultured only with spheroids, detailed evaluation of individual effector functions combinations evidenced that, CD107a+IFN-γ+ population was reduced and CD107a+TNF-α+ and IFN-γ+TNF-α+ subsets were increased in control NK cells (online **Supplementary Figure 4B**). Similarly, although there was no statistical significance in polyfunctionality enhancement when exposing pre-CIML NK cells to tumor spheroids (**Figure 1C**), there was a significant increase in triple-positive cells, as well as in the double-positive CD107a+IFN-γ+ and IFN-γ+TNF-α+ cells (online **Supplementary Figure 4B**). This indicates that the effector functions of pre-CIML NK cells are improved when exposed to tumor spheroids. Polyfunctional profiling indicated that IL-12/15/18-stimulation strongly induced IFN-γ production, while cetuximab primarily drove TNF-α production, and both stimuli led to enhanced degranulation (**Figure 1C**).

Altogether, these results demonstrate that NK cells are responsive to both lung cancer and CRC spheroids, and their activation and anti-tumor cytokine production are enhanced with IL-12/15/18 stimulation, as well as by ADCC induction.

### Pre-CIML NK cells exhibit increased cytotoxicity against tumor spheroids, which is further enhanced by ADCC induction

**Figure 2:**
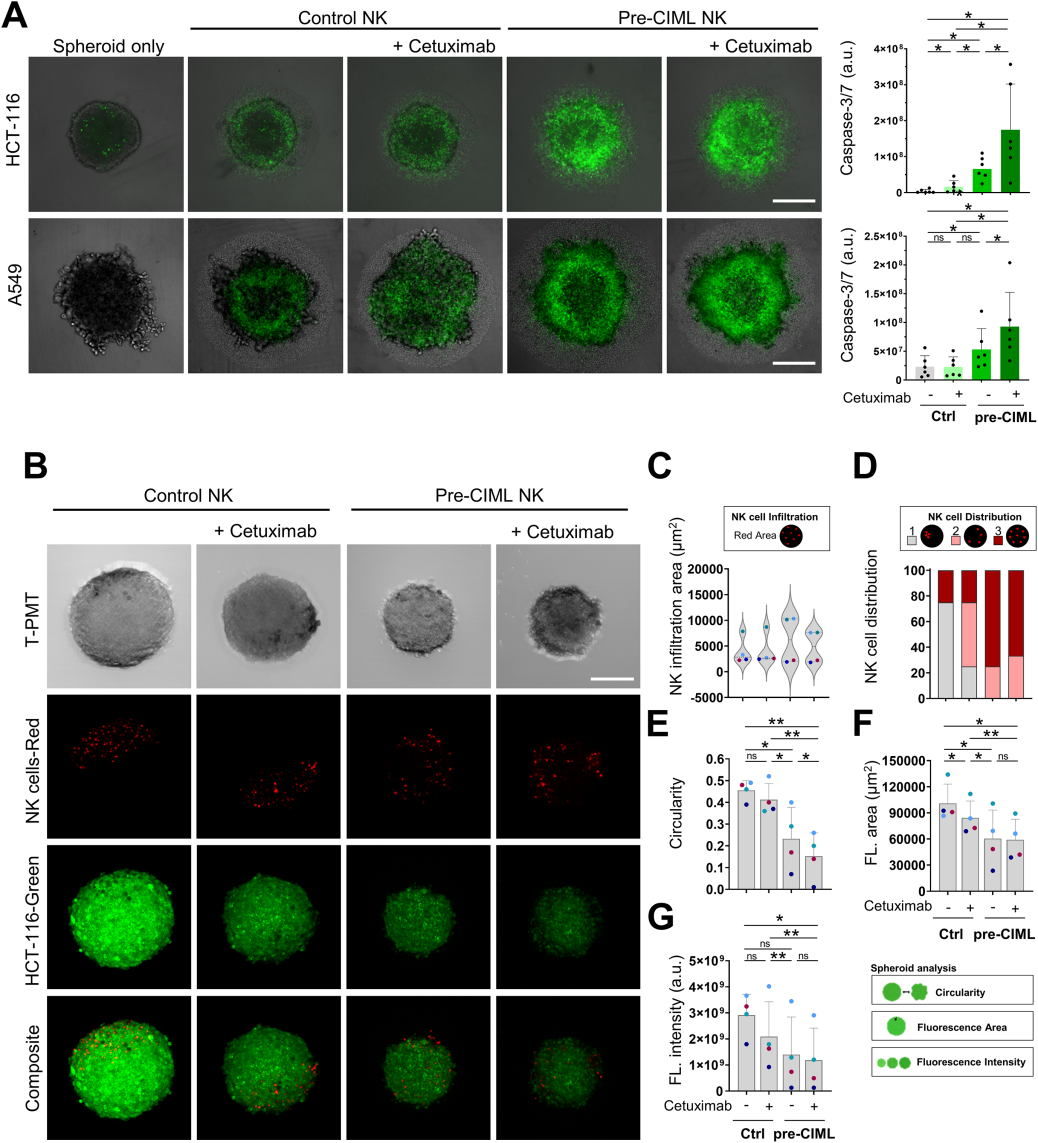
NK cell cytotoxicity and infiltration in spheroids. **(A)** Unlabeled control or pre-CIML NK cells were co-cultured (5:1 E:T ratio) with HCT-116 (top) and A549 (bottom) tumor spheroids in the presence or absence of 1 µg/mL cetuximab. Representative overlay of transmitted light and Caspase-3/7 green images after 24 hours of co-culture (left) and bar graphs showing total Caspase-3/7 green fluorescence quantification (right). Scale bar 300 μm. **(B–G)** NK cells labelled with eFluor™ 670 (red) were co-cultured (3:1 E:T ratio) with HCT-116-Green spheroids for 20 hours, in the presence or absence of 1 µg/mL cetuximab. **(B)** Representative images of the co-culture acquired in T-PMT and fluorescence: NK cells (red), spheroid (green), and merged images (composite). Scale bar 150 μm. **(C)** Violin plots showing NK cell infiltration as the red fluorescent area within the spheroid. **(D)** Spatial distribution of red fluorescence by scoring (1, infiltration confined to a single region; 2, infiltration into multiple regions with a non-uniform, scattered distribution; 3, uniform distribution throughout the spheroid). Stacked bar graph showing the percentage of spheroids assigned to each spatial distribution score. Bar graphs show the mean ± SD with color-coded donors showing spheroid´s circularity **(E)**, area **(F)** and total intensity **(G)** of green fluorescence. Bar graphs show the mean ± SD of 4 or 6 independent donors. Statistical analyses used paired t-tests and significance values are indicated as *(p<0.05) and **(p<0.01). ns: non-significant.

Having established that pre-CIML NK cells display superior degranulation and cytokine production in response to tumor spheroids, we next evaluated their cytotoxic potential. HCT-116 and A549 spheroids were co-cultured for 24 hours with either control or pre-CIML NK cells derived from six independent donors, and cell death was assessed by caspase-3/7 detection. Control NK cells induced only low levels of caspase-3/7 activation in both HCT-116 and A549 spheroids. Conversely, pre-CIML NK cells exhibited a significantly improved killing capacity against both tumor spheroids. The addition of cetuximab, further enhanced pre-CIML NK cell-mediated cytotoxicity, and to a lesser extent, increased cytotoxicity of control NK cells only when cultured with HCT-116 spheroids (**Figure 2A**). These findings demonstrate that pre-CIML NK cells effectively kill tumor cells growing as 3D spheroid cultures, and this cytotoxicity can be further potentiated with cetuximab-mediated ADCC.

### Pre-CIML NK cells show different intratumoral distribution and compromise spheroid integrity

To determine whether the enhanced cytotoxicity of pre-CIML NK cells was associated with increased access into tumor spheroids, we co-cultured fluorescently labelled NK cells from four independent donors with HCT-116-Green spheroids. After 20 hours, spheroids were collected, transferred to chamber slides, and imaged by confocal microscopy to visualize NK cells within the spheroid (**Figure 2B**). The overall area of infiltrating NK cells was comparable across conditions (**Figure 2C**), and represented only a small fraction within the spheroid, suggesting that the Caspase-3/7 signal measured in **Figure 2A** predominantly reflected apoptosis of tumor cells. Interestingly, their spatial distribution differed: control NK cells were predominantly confined to a peripheral region of the spheroid, while pre-CIML NK cells displayed a more uniform distribution throughout the spheroid (**Figure 2B**). To further explore this, we assessed NK cell distribution and confirmed that pre-CIML NK cells indeed exhibited a more homogeneous intratumoral pattern (**Figures 2B, D**), whereas cetuximab treatment did not have a significant effect on NK cell spatial distribution. This suggests that pre-CIML NK cells may possess superior infiltrative capacity within 3D tumor systems, which could contribute to their enhanced cytotoxicity.

Pre-CIML NK cells also induced pronounced structural disruption of spheroids (**Figure 2B**). Image-based analyses showed that spheroids co-cultured with pre-CIML NK cells were smaller and exhibited reduced circularity compared to those exposed to control NK cells(**Figures 2B, E**; online **Supplementary Figure 5**). Furthermore, a marked loss of green fluorescence (HCT-116-Green) in spheroids treated with pre-CIML NK cells suggested compromised viability, consistent with their enhanced cytotoxic potential (**Figures 2F, G**; online **Supplementary Figure 5**). This approach aligns with a previous study demonstrating that loss of GFP fluorescence correlates with reduced cell viability in SH-SY5Y spheroids (40). These effects likely reflect both the enhanced cytotoxic capacity and the better intratumoral distribution of pre-CIML NK cells within the spheroid, allowing deeper and more effective engagement of tumor cells. Cetuximab-mediated ADCC subtly contributed to spheroid disruption (**Figures 2E, F, G**).

### Pre-CIML NK cells show enhanced anti-tumoral functions against patient-derived CRC organoids

**Figure 3:**
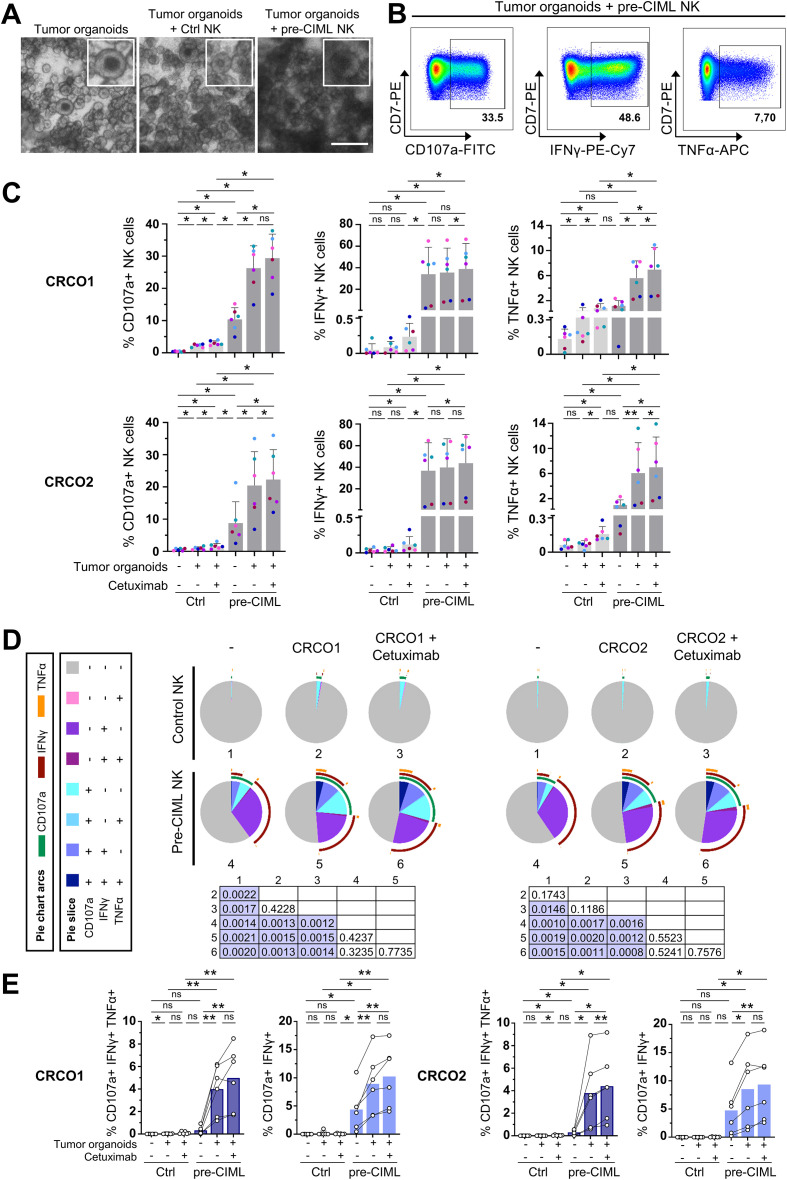
NK cell degranulation and cytokine production in response to tumor organoids. Control or pre-CIML NK cells were co-cultured (2:1 E:T ratio) with tumor organoids (CRCO1 and CRCO2) for 5 hours in the presence or absence of 1 µg/mL cetuximab. **(A)** Representative transmitted light images of the co-culture with CRCO1 tumor organoids. Scale bar 300 µm. **(B)** Representative flow cytometry pseudocolor plots showing the percentage of pre-CIML NK cells expressing CD107a, IFN-γ or TNF-α after the co-culture. **(C)** Bar graphs showing the percentages of NK cells expressing CD107a, IFN-γ or TNF-α after co-culture with CRCO1 (top), or CRCO2 (bottom) organoids. Bar graphs show the mean ± SD of color-coded donors (n=6). Statistical analysis was performed using Wilcoxon tests and significance is indicated as *(p<0.05) and **(p<0.01). ns: non-significant. **(D)** Pie charts depicting the proportions of NK cells expressing CD107a, IFN-γ and/or TNF-α in the indicated culture conditions. The slices indicate cells expressing different combinations of markers and the arcs denote which individual marker they express. Differences between conditions were assessed with a non-parametric permutation test (n=6) and p-values are shown in the matrix. Significant differences are highlighted in purple. **(E)** Bar graphs showing the frequency of polyfunctional NK cells co-expressing CD107a, IFN-γ and TNF-α (dark blue) or CD107a and IFN-γ (light blue). Statistical analysis was performed using paired t-tests and significance is indicated as *(p<0.05) and **(p<0.01). ns: non-significant.

To extend our observations to more physiologically relevant models, we next evaluated NK cell responses in patient-derived CRC organoids, which better recapitulate the genetic and structural features of human tumors. Two patient-derived CRC organoids (CRCO1 and CRCO2) were used, differing in morphology, ligand surface expression and genetic mutation profile, including *KRAS* mutation status (41) (online **Supplementary Figure 3**; online **Supplementary Tables 1, 2**). Visually, co-culture with control NK cells had minimal impact on CRCO viability, while pre-CIML NK cells caused evident disruption of organoid structures, appearing less defined, with visible debris, and general disintegration (**Figure 3A**), suggesting cytotoxic activity. NK cell function was quantified by flow cytometry, measuring NK cell degranulation and cytokine production (**Figure 3B**). Control NK cells displayed low baseline frequencies of CD107a+ cells, with a modest but significant increase upon organoid exposure (**Figure 3C**). In contrast, pre-CIML NK cells exhibited a higher basal frequency of CD107a+ cells, which was further enhanced upon co-culture with both CRCO1 and CRCO2 organoids. Regarding cytokine production, the frequency of IFN-γ+ control NK cells was very low, which did not increase upon organoid exposure (**Figure 3C**). Pre-CIML NK cells, as expected, showed a significantly higher proportion of IFN-γ producing cells at baseline, which slightly increased after co-culture. Regarding TNF-α, control NK cells showed low basal frequency that increased only in response to CRCO1 organoids, while pre-CIML NK cells displayed higher basal TNF-α+ frequencies that were further elevated upon co-culture with both organoid lines (**Figure 3C**). These trends were consistent with MFI data (online **Supplementary Figure 6**). Together, these data confirm that NK cells respond to patient-derived tumor organoids and that IL-12/15/18-preactivation enhances their effector functions.

We proceeded to evaluate whether cetuximab-induced ADCC could also enhance NK cell responses in these 3D systems. Despite the low expression of EGFR (online **Supplementary Figure 3**), the addition of cetuximab modestly but significantly increased the frequencies of CD107a+ and TNF-α+ subsets in control NK cells co-cultured with tumor organoids, although the increase was non-significant for IFN-γ+ subsets (**Figure 3C**). In pre-CIML NK cells, cetuximab also led to modest increases in the frequencies of CD107a+, TNF-α+, and IFN-γ+ cells, with increases similar in magnitude to those in control NK cells. Overall, cetuximab-driven enhancement of NK cell activity in the organoid system was relatively modest and less pronounced than in 3D spheroid models.

Polyfunctionality analysis provided an integrated view of these responses. Control NK cells remained largely non-functional, with only a slight increase in polyfunctionality upon CRCO1 organoid exposure that slightly improved in the presence of cetuximab (**Figure 3D**). In contrast, pre-CIML NK cells were more functional, with a marked increase in IFN-γ production. Co-culturing pre-CIML NK cells with organoids slightly increased the polyfunctionality, and the effect of cetuximab was minimal (**Figure 3D**). However, detailed evaluation of individual functional combinations evidenced a significant increase in the triple-positive (CD107a+TNF-α+IFN-γ+) and CD107a+IFN-γ+ populations (**Figure 3E**) in pre-CIML NK cells when exposed to both tumor organoids. Conversely, when assessing the effect of cetuximab, we found no significant differences in pre-CIML NK cells co-cultured with tumor organoids (**Figure 3D**). Nonetheless, it had a significant effect in the frequency of triple-positive pre-CIML NK cells in CRCO2 organoids (**Figure 3E**). Altogether, pie chart analysis showed that cytokine stimulation is the major driver of NK cell functionality in the organoid system, with a slight contribution of cetuximab-mediated ADCC.

### Pre-CIML NK cells exhibit increased cytotoxic activity against patient-derived CRC organoids

**Figure 4:**
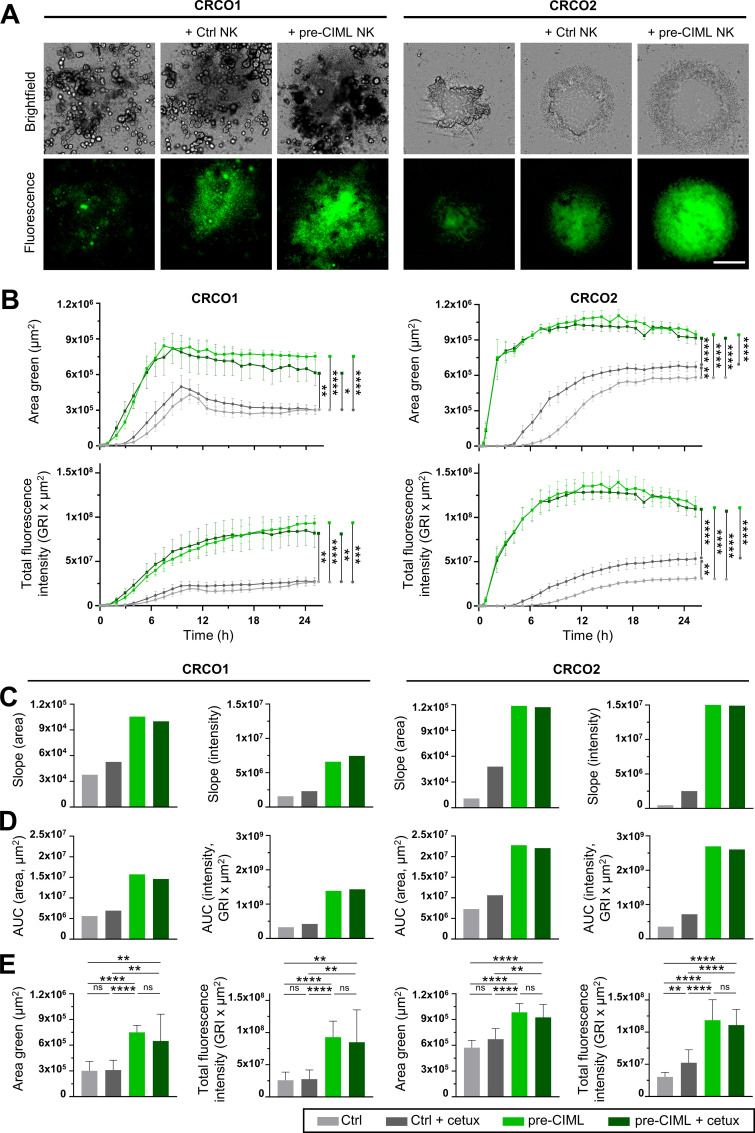
NK cell cytotoxicity against tumor organoids. Control or pre-CIML NK cells were co-cultured (5:1 E:T ratio) with tumor organoids (CRCO1, left and CRCO2, right) in the presence or absence of 1 µg/mL cetuximab. **(A)** Representative brightfield and caspase-3/7 green fluorescence images at 24 hours of culture. Scale bar 500 µm. **(B)** Time course graphs showing the caspase-3/7 area (top) and intensity (bottom) for each culture condition: ctrl NK cells without (light gray) or with cetuximab (dark gray); and pre-CIML NK cells without (light green) or with cetuximab (dark green). Curves show mean ± SEM from 3 independent donors in technical triplicates. Statistical comparisons were performed by two-way Anova test and significance is indicated as *(p<0.05), **(p<0.01), ***(p<0.001) and ****(p<0.0001). From the mean of the curves in **(B)**, the slope (0–9 hours, **(C)**) and the area under the curve [AUC, **(D)**] are shown as single integrated values. **(E)** Fluorescence area and fluorescence total intensity at 24 hours after the co-culture. **(C–E)** Bar graphs show the mean ± SD from 3 independent donors in technical triplicates. **(E)** Statistical analysis was performed using unpaired t-tests and statistical significance values are indicated as **(p<0.01), ***(p<0.001) and ****(p<0.0001). ns: non-significant.

We next assessed the cytotoxic potential of NK cells in the 3D organoid co-cultures using real-time imaging with the xCELLigence RTCA eSight system. Despite morphological differences between the organoid lines, both showed a similar pattern of cell death after 24 hours of co-culture. Organoids cultured alone exhibited minimal caspase-dependent cell death, whereas co-culture with control NK cells resulted in increased cell death that was further enhanced in the presence of pre-CIML NK cells, which also caused marked disruption of organoid morphology (**Figure 4A**; online **Supplementary Figures 7**, **8**).

Quantification of the apoptotic area and the total apoptotic fluorescence intensity over time revealed that control NK cells required more time to initiate killing, whereas pre-CIML NK cells triggered rapid and robust cell death soon after co-culture initiation, being this effect more pronounced in CRCO2 (**Figure 4B**). Kinetic analysis of the slopes of caspase-3/7 activation over time confirmed these observations (**Figure 4C**). Closer evaluation of tumor-death onset showed that control NK cells initiated killing earlier in CRCO1 (around 3 hours after co-culture start), while in CRCO2 the apoptotic signal appeared later (approximately 6 hours). ADCC-induction with cetuximab accelerated control NK cell killing in CRCO2 and maintained higher levels of caspase-dependent cell death throughout the co-culture (**Figures 4B, C**). In contrast, cetuximab did not enhance control NK cell killing in CRCO1, nor did it improve the potent cytotoxicity of pre-CIML NK cells in either organoid model. These findings were consistent with AUC analysis (**Figure 4D**) as well as endpoint measurements at 24 hours (**Figure 4E**). Together, they show that control NK cells can mediate tumor cell death, and that this effect is potentiated by ADCC, but only in specific contexts (i.e., CRCO2 organoids). Preactivation with IL-12/15/18 dramatically enhanced NK-mediated killing in both CRCO1 and CRCO2 organoids, and this potent cytotoxic activity was not further improved by cetuximab.

## Discussion

NK cells are key players in antitumor immunity, and NK cells stimulated with IL-12/15/18 exhibit enhanced effector functions linked to metabolic, transcriptional, and epigenetic adaptations (9, 19, 22, 23, 42, 43). Several studies have described that upon stimulation, these NK cells undergo alterations in the expression of activating and inhibitory receptors, such as CD16 downregulation and NKG2A and TIM-3 upregulation, with a consistent overall increase in anti-tumor effector functions (44, 45). Despite the success of IL-12/15/18-stimulated NK cells in hematological malignancies (18, 21), their efficacy in solid tumors remains limited. Here, we show that pre-CIML NK cells exert potent anti-tumor activity against solid tumor models, including lung and CRC spheroids and patient-derived CRC organoids. Cetuximab further augmented NK cell effector functions, although its impact was context-dependent: robust in spheroids but more modest in organoids. Together, our findings support the therapeutic potential of combining pre-CIML NK cells with cetuximab to improve immunotherapy against solid tumors.

IL-12/15/18 cytokines induced a marked increase in NK cell polyfunctionality against HCT-116 and A549 spheroids, with pronounced gains in IFN-γ production. These results extend cell-based observations to more complex 3D tumor models, consistent with prior works showing that cytokine preactivation enhances NK cell effector functions against target cells (19, 23) and it aligns with improved NK cell degranulation reported in CRC spheroids after cytokine priming (46). The superior cytotoxicity of pre-CIML NK cells displayed in both lung and CRC spheroids, relative to control NK cells, agrees with reports in CRC (33) and gastric cancer (31) spheroid models, indicating that short stimulation with IL-12/15/18 drives functional enhancement that translates into effective tumor killing even within the physically restrictive context of 3D tumors.

NK cell infiltration in solid tumors correlates with improved patient survival (47), yet limited immune cell access remains a major barrier in cancer immunotherapy (24, 25). 3D tumor models provide a relevant platform to study this process, as they recapitulate tumor architecture and microenvironmental barriers. In our tumor spheroid model, control NK cells exhibited low infiltration and were predominantly localized at the spheroid periphery, consistent with observations in gastric (31), cervical (30) and CRC (46) tumor spheroids. Although some studies have evaluated infiltration of activated NK cells (30, 32), most lack direct comparison with non-activated controls, making it difficult to assess true improvements in infiltration. Interestingly, in a gastric cancer spheroid model, CIML NK cells were directly compared to control NK cells and showed a modest but measurable improvement in tumor infiltration (31). In line with this, although we did not observe an increase in the overall number of infiltrating cells at 20 hours of co-culture, pre-CIML NK cells displayed a more homogeneous intratumoral distribution, suggesting that cytokine priming enhances intratumoral navigational capacity and may help overcome structural constraints within solid tumors. In pre-CIML NK cells, we did not detect an increase in the expression of chemokine receptors and adhesion molecules (CXCR1/2/3/4, CD103 and CD62L) (online **Supplementary Figure 9**), suggesting that these do not play a direct role in NK cell migration in this experimental setting, where physical contact between NK cells and spheroids is already facilitated. Instead, we observed an increase in CD49a, which may potentially contribute to NK cell motility within tumor spheroids in our system due to its capability of interacting with extracellular matrix components (48). Of note, NK cell infiltration has been reported to be more evident at low E:T ratios and to be highly dynamic (32), which, hence, can contribute to the variability in NK cell infiltration across studies. Therefore, implementing time-course analyses and evaluating different E:T ratios will be important to fully understand and improve NK cell infiltration within solid tumors.

Patient-derived organoids are becoming increasingly relevant for *in vitro* studies, as they not only recapitulate key architectural features of tumors but also retain patient-specific histological and genetic features, making them a valuable platform for therapeutic testing (28, 29, 49–52). While our model does not incorporate additional TME components, such as cancer-associated fibroblasts or other immune cells that may contribute to immunosuppression, this 3D system allows us to accurately assess the direct NK cell activity against tumor cells in phenotypically and morphologically heterogeneous patient-derived tumor 3D cultures (53–55). In both CRCO1 (RAS wild-type) and CRCO2 (RAS-mutant) organoids, pre-CIML NK cells showed a clear increase in degranulation and cytokine production compared to control NK cells. This further confirms that IL-12/15/18 preactivation strongly enhances polyfunctionality regardless of whether the model is based on 2D or 3D culture systems (19, 23, 31, 33).

NK cell-mediated cytotoxicity in tumor organoid models remains relatively understudied. Previous work has shown that IL-2-activated NK cells can kill pancreatic tumor organoids at high E:T ratios (52), and that HER2-specific CAR-NK cells reduce breast cancer organoid growth in a time and organoid size dependent manner (56). However, most studies rely on endpoint measurements or dissociated organoids, limiting the understanding of the real-time dynamics of NK cell activity in whole tumor organoids. Here, we used the xCELLigence real-time cell analysis system, which is widely used for impedance-based monitoring of cellular behavior, to dynamically monitor NK cell-organoid interactions in real time. Based on the available literature, this is the first study combining this platform with whole, intact tumor organoids, enabling continuous cytotoxicity monitoring while preserving their 3D architecture. Furthermore, to the best of our knowledge, this is also the first study assessing the cytotoxicity of pre-CIML NK cells against patient-derived tumor organoids. Real-time analysis revealed a markedly enhanced cytotoxic response of pre-CIML NK cells against both organoid lines, leading to faster and more efficient tumor cell killing compared to control NK cell, indicating that the functional enhancement of pre-CIML NK cells translated into markedly improved cytotoxicity against organoids.

Cetuximab (anti-EGFR mAb) is clinically used to treat *RAS* wild-type CRC and HNSCC, and previous *in vitro* studies have shown that, although cetuximab alone may exert minimal cytotoxicity (57, 58), its combination with NK cells enhances tumor cell killing through ADCC (59). This has been shown with other mAbs, such as trastuzumab (anti-HER-2 mAb) (52, 60) and avelumab (anti-PD-L1 mAb) (52). Therefore, in this work we investigated whether the combination of cetuximab with pre-CIML NK cells could induce more robust anti-tumoral responses in 3D tumor models. In our spheroid models, cetuximab markedly enhanced NK cell polyfunctionality and cytotoxicity in both control and pre-CIML NK cells. Its strong enhancement of TNF-α production complemented the IFN-γ-driven response of pre-CIML NK cells, yielding the highest overall polyfunctional profile. These data highlight the therapeutic potential of combining adoptive cell therapy of pre-CIML NK cells with cetuximab to treat patients with CRC and HNSCC (see NCT05674526).

In contrast to the strong cetuximab-mediated ADCC responses observed in spheroids, organoids showed only modest improvements in NK cell effector functions, which could be due to their lower EGFR surface expression. While ADCC has been reported to occur independently of ligand expression levels in pancreatic cancer organoids (52), a minimal EGFR expression threshold may still be required to trigger effective cetuximab-mediated ADCC in our system. The slight cetuximab-driven increase in control NK cell-mediated cytotoxicity in *KRAS*-mutant CRCO2 indicates that ADCC acts independently of *KRAS* status and functions as an immune engager, consistent with reports of IL-2/15-activated NK cells mediating ADCC regardless of *RAS/BRAF* mutations (61, 62).

Notably, CRCO2 expresses lower surface levels of HLA class I compared to CRCO1, which could partially explain the enhanced cetuximab-mediated killing of CRCO2 by control NK cells. Nevertheless, previous reports have demonstrated that the inter-donor heterogeneity impacts organoid susceptibility to NK cell-mediated killing, as well as ADCC (51, 52, 63). Hence, given the considerable genetic and morphological heterogeneity between the two organoids, it is likely that multiple mechanisms contribute to the observed differences in cytotoxicity, reflecting a combination of architectural and tumor-intrinsic features rather than a single dominant factor. For instance, the compact epithelial architecture of CRCO2 may restrict antibody penetration and NK cell access, thereby limiting ADCC efficiency (64). Further studies with larger organoid panels will be necessary to dissect the relative contribution of each factor and to identify predictive biomarkers of organoid susceptibility to NK cell killing, including cetuximab-mediated ADCC.

In summary, our study extends prior evidence of pre-CIML NK activity into complex 3D models of solid tumors, including spheroids and patient-derived organoids. IL-12/15/18 preactivation endowed NK cells with robust anti-tumor activity across CRC and lung cancer 3D models, enhancing degranulation, cytokine production, cytotoxicity, and intratumoral distribution. While cetuximab-mediated ADCC further augmented these responses in spheroids, it provided limited benefit in organoids, suggesting how tumor architecture and model complexity can influence ADCC efficacy. These findings support the therapeutic potential of pre-CIML NK cells in solid tumors and suggest that their combination with therapeutic mAbs that leverage ADCC (e.g., cetuximab) may be advantageous in selected contexts. They also highlight the value of patient-derived organoids as physiologically relevant platforms for evaluating NK cell-based therapies. Future studies incorporating larger organoid panels and *in vivo* systems will be important to further define the translational potential of this approach for the treatment of solid tumors.

## Data Availability

The raw data supporting the conclusions of this article will be made available by the authors, without undue reservation.
