# Supplementary material for: Cytokine preactivation and ADCC: a potent strategy for enhancing human NK cell effector functions against 3D tumor models
Source: Front Immunol. 2026 May 8;17:1797290. doi: 10.3389/fimmu.2026.1797290 (PMC13194526; doi:10.3389/fimmu.2026.1797290)
Supplement: Supplementary file 1 [file DataSheet1.pdf]

Supplemental Information for

Cytokine preactivation and ADCC: a potent strategy for enhancing human NK cell effector functions against 3D tumor models

This file includes:

- Supplemental figure 1. Schematic representation of the experimental workflow.
- Supplemental figure 2. Gating strategy to analyze NK cell degranulation and cytokine production in response to tumor spheroids or organoids.
- Supplemental figure 3. Ligand expression in the tumor models used in this study.
- Supplemental figure 4. Degranulating and cytokine producing NK cell populations in response to spheroids.
- Supplemental figure 5. NK cell-mediated disruption of tumor spheroids.
- Supplemental figure 6. MFI of NK cell degranulation marker CD107a and cytokine production in response to organoids.
- Supplemental figure 7. Representative images of NK cell-mediated cytotoxicity against patient-derived CRCO1 organoid.
- Supplemental figure 8. Representative images of NK cell-mediated cytotoxicity against patient-derived CRCO2 organoid.
- Supplemental figure 9. Expression of chemokine receptors and adhesion molecules on NK cells.
- Supplemental table 1. Mutational status of tumor models.
- Supplemental table 2. Mutational profile of tumor organoids.

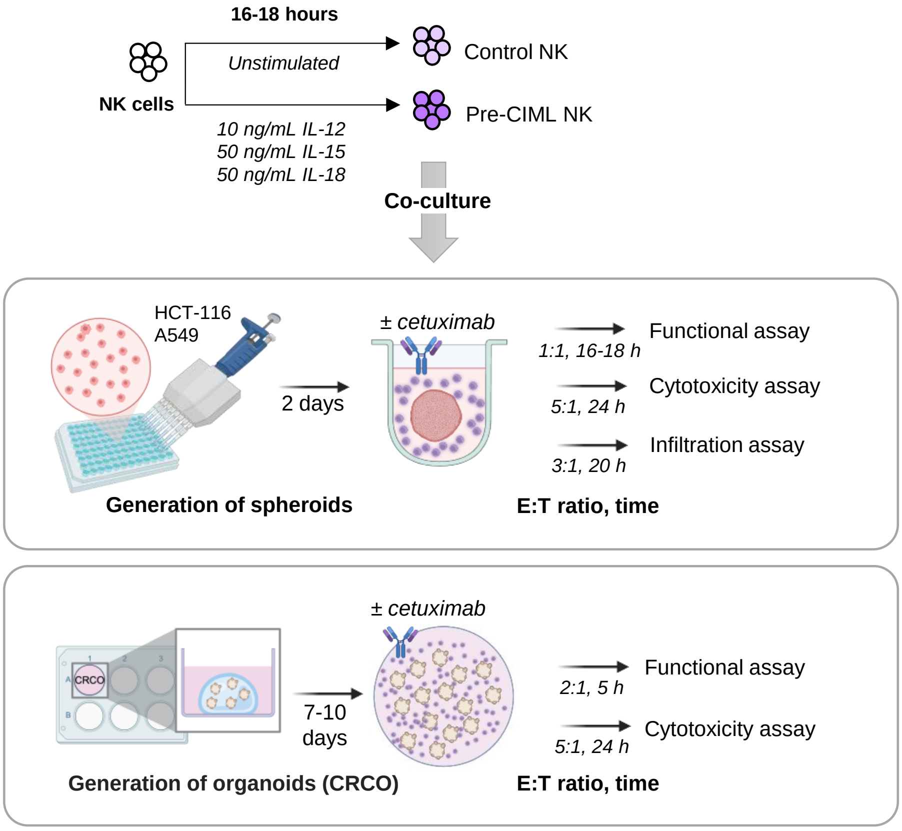

Supplemental figure 1. Schematic representation of the experimental workflow. NK cells were cultured for 16–18 hours at 37°C with 10 ng/mL IL-12, 50 ng/mL IL-15 and 50 ng/mL IL-18, or in media alone to obtain pre-CIML NK cells and control NK cells, respectively. Prior to co-culture assays with NK cells, tumor models were generated. For spheroid formation, tumor cells (A549 and HCT-116) were seeded on round-bottom ultra-low attachment 96-well plates and were allowed to grow for two days until a mature spheroid was formed per well. Organoids (CRCOs) were maintained in culture for 7–10 days to allow their progression from single cells/small aggregates to fully developed organoid cultures, when they were collected and transferred to the appropriate plates for co-culture assays. Control or pre-CIML NK cells were then added to mature spheroids or organoids in the presence or absence of 1 µg/mL cetuximab. Co-culture duration and E:T ratios were adjusted for the requirements of each tumor model and objective of the assay, and are indicated in the diagram. Created in BioRender. Lopez, A. (2026) https://BioRender.com/3gfwg63.

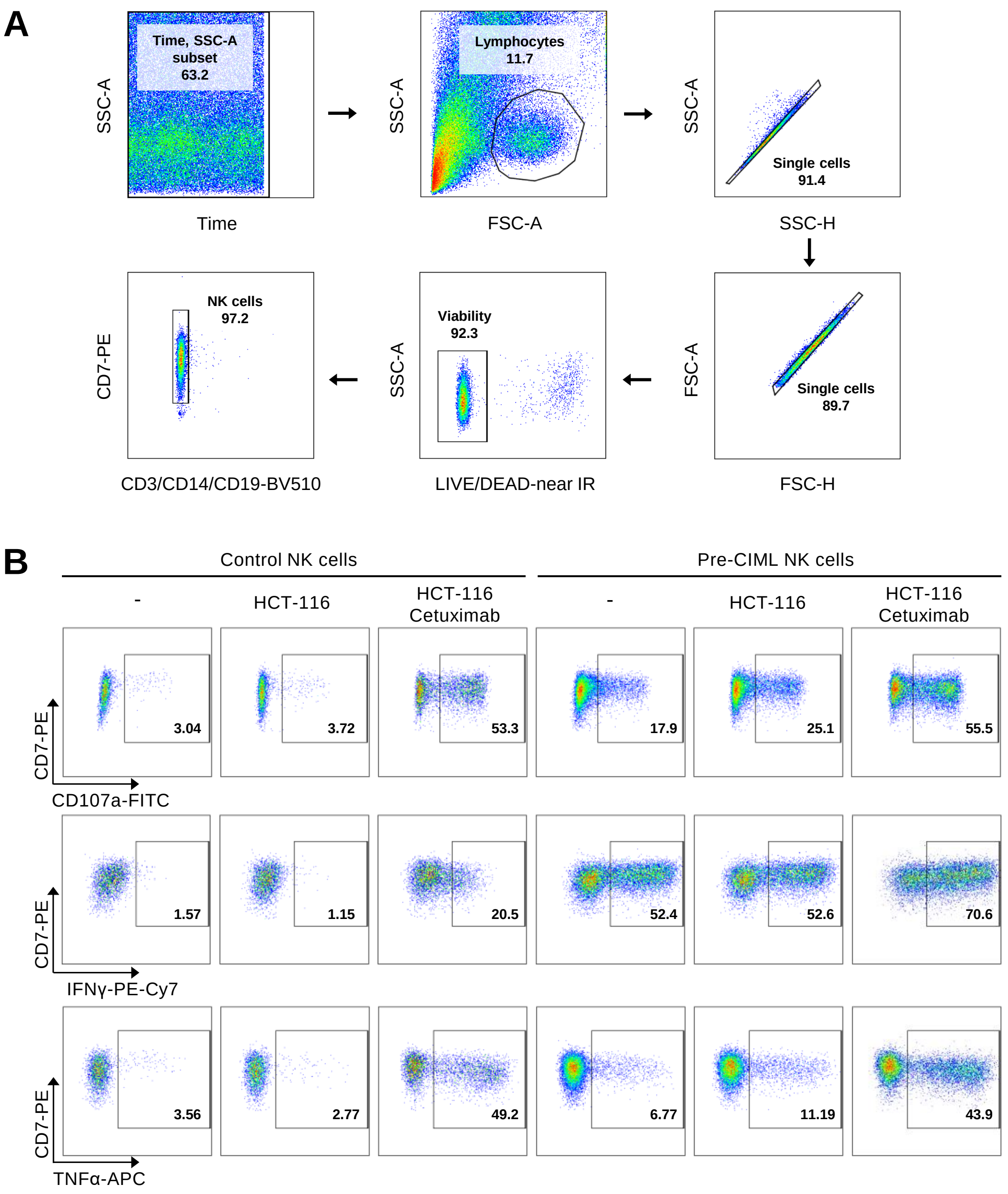

Supplemental figure 2. Gating strategy to analyze NK cell degranulation and cytokine production in response to tumor spheroids or organoids. (A) Representative pseudocolor plots illustrating the gating strategy for identifying NK cells. First, irregular acquisition events were excluded using a time gate (Time vs. SSC-A). Then, lymphocytes were selected based on the forward and side scatter area (FSC-A vs. SSC-A). Next, singlets were defined by two consecutive doublet exclusions: SSC-A vs. SSC-H followed by FSC-A vs. FSC-H. Dead cells were then excluded by gating on LIVE/DEAD negative cells. Finally, NK cells were defined as CD3−CD14−CD19−CD7+. **(B)** Representative pseudocolor plots showing the frequencies of control and pre-CIML NK cells expressing CD107a, IFN-γ or TNF-α after exposure to HCT-116 spheroids with or without cetuximab.

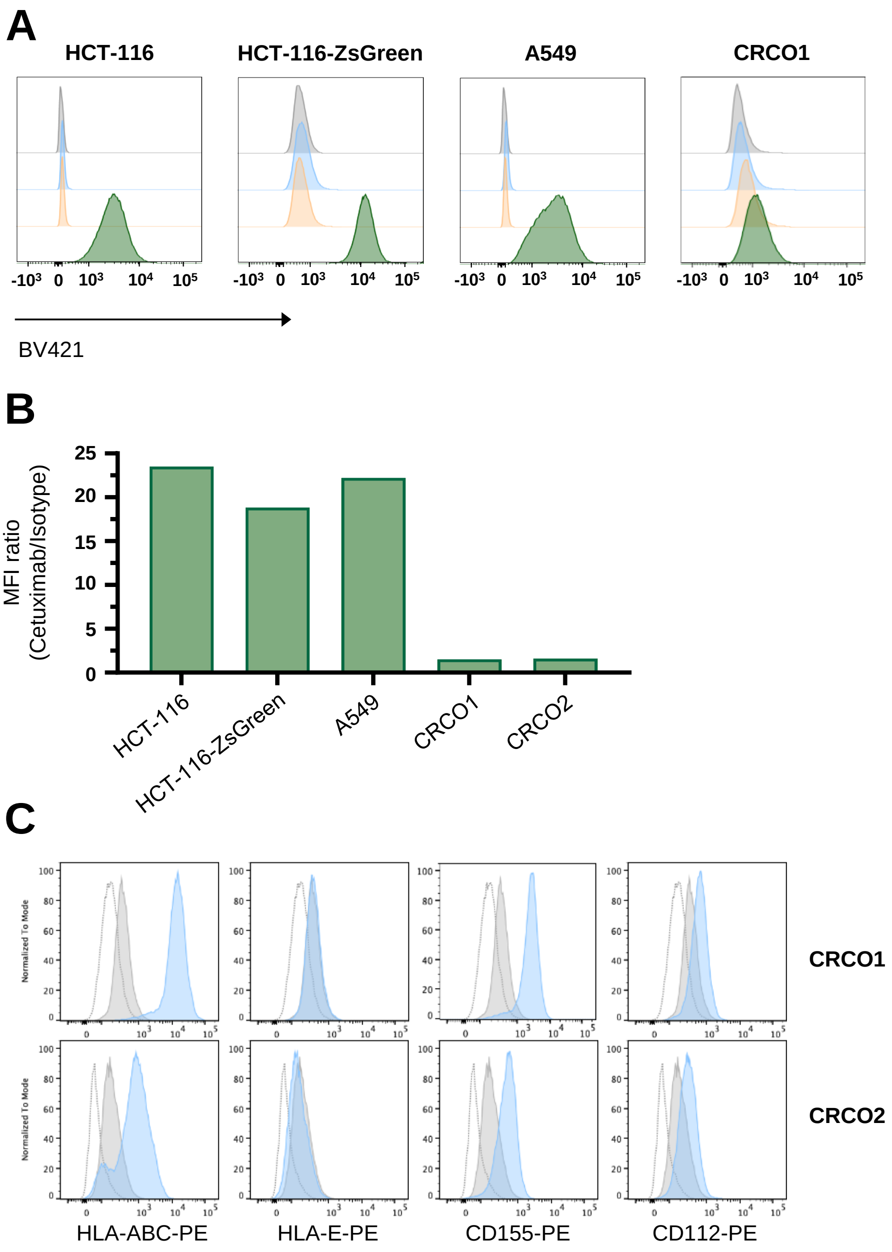

Supplemental figure 3. Ligand expression in the tumor models used in this study. (A) EGFR expression on HCT-116, HCT-116-Green, and A549 cell lines, and on CRCO1 and CRCO2 organoids, was assessed by flow cytometry using cetuximab (green) followed by a secondary antibody (donkey anti-human IgG BV421-conjugated). Controls included rituximab plus secondary antibody (orange), secondary antibody alone (blue), and unstained cells (gray). **(B)** Relative EGFR surface expression quantified as the ratio of the mean fluorescence intensity (MFI) of cetuximab-stained to rituximab-stained samples (cetuximab/isotype). **(C)** Surface expression of HLA-ABC (PE anti-human IgG1, clone G46-2.6, BD Biosciences), HLA-E (PE anti-human IgG1, clone 3D12, Biolegend), CD155 (PE anti-human IgG1, clone SKII.4, Biolegend) and CD112 (PE anti-human IgG1, clone TX31, Biolegend) in both tumor organoids (blue). Isotype controls (grey) and unstained controls (grey line) are also included.

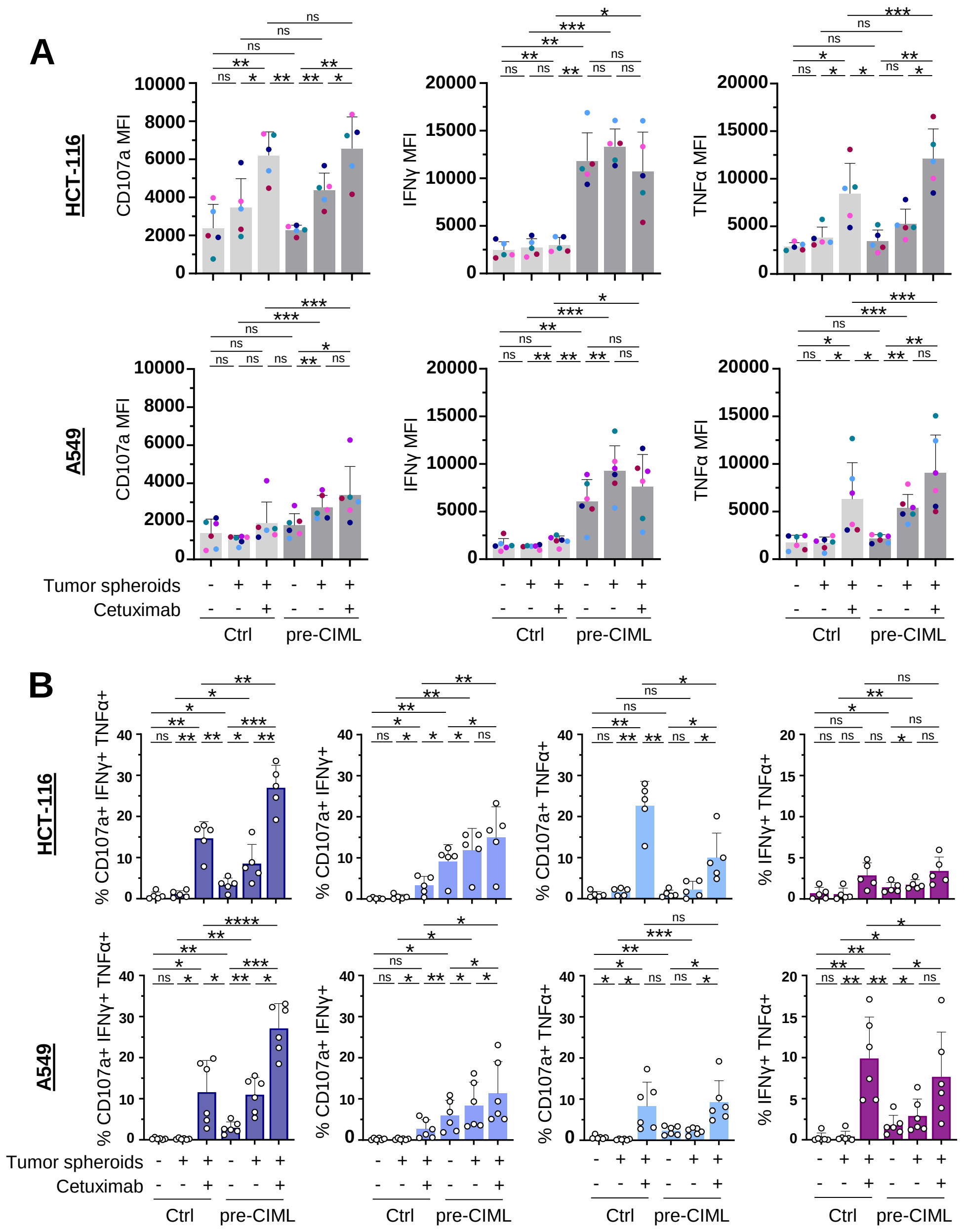

Supplemental figure 4. Degranulating and cytokine producing NK cell populations in response to spheroids. Control or pre-CIML NK cells were co-cultured with tumor spheroids (A549 and HCT-116) at 1:1 E:T ratio for 16-18 hours, in the presence or absence of 1 µg/mL cetuximab. **(A)** Bar graphs showing the MFI of CD107a, IFN-γ and TNF-α within the respective CD107a+, IFN-γ+ or TNF-α+ NK cell populations. Donor color-coded data points. **(B)** Bar graphs display the frequencies of NK cells co-expressing CD107a, IFN-γ and TNF-α (dark blue); CD107a and IFN-γ (medium blue); CD107a and TNF-α (light blue); and IFN-γ and TNF-α (purple). These populations correspond to those represented in the polyfunctionality pie charts shown in figure 1C. Each dot represents one donor. For **(A)** and **(B)** data are mean ± SD of 5 independent donors for HCT-116 spheroids and 6 for A549 spheroids. Statistical analyses were performed using paired t-tests, and significance values are indicated as *(p<0.05), **(p<0.01), ***(p<0.001), and ****(p<0.0001).

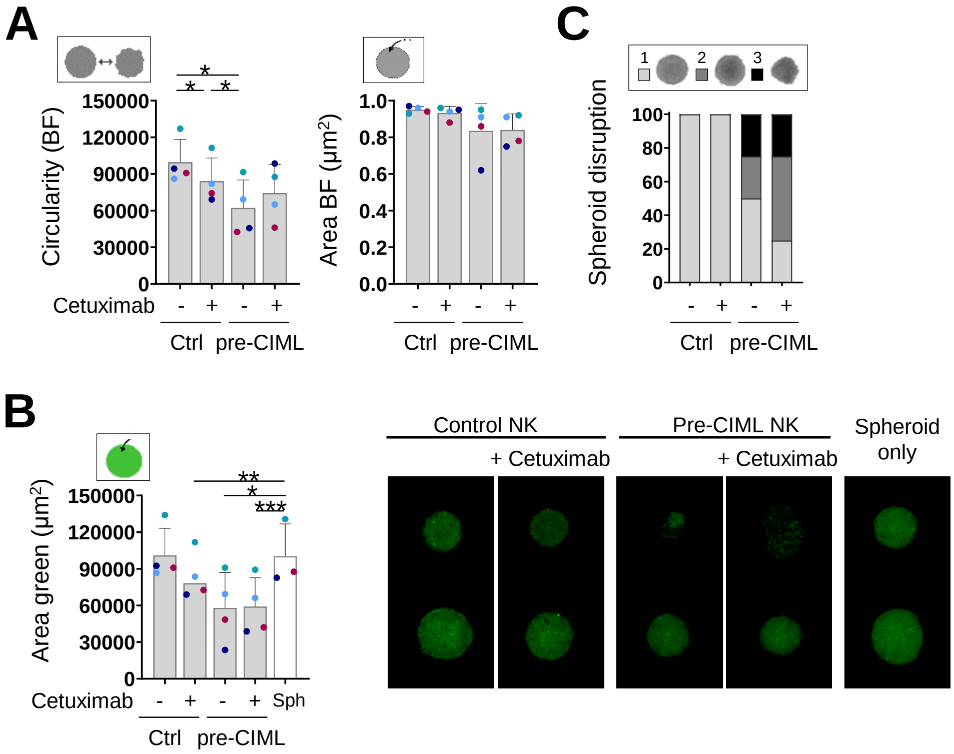

Supplemental figure 5. NK cell-mediated disruption of tumor spheroids. Control or pre-CIML NK cells, were co-cultured at 3:1 E:T ratio with HCT-116-Green spheroids for 20 hours, in the presence or absence of 1 µg/mL cetuximab. **A)** Spheroid disruption was also assessed in the transmitted light images (T-PMT) by loss of spheroid circularity and decreased area. **B)** The spheroid green fluorescence area was measured for all the conditions including spheroid-only (Sph) controls. Representative images are shown. Bar graphs show mean ± SD with color-coded donors, and statistical analysis was performed using paired t-test indicating significance values as: *($p<0.05$), **($p<0.01$), ***($p<0.001$). **C)** In addition, T-PMT images were randomized and scored on a 1–3 scale for structural disruption: 1, spheroid with preserved integrity; 2, partial damage; 3, loss of structural integrity. The stacked bar graph shows the percentage of spheroids assigned to each spatial distribution score.

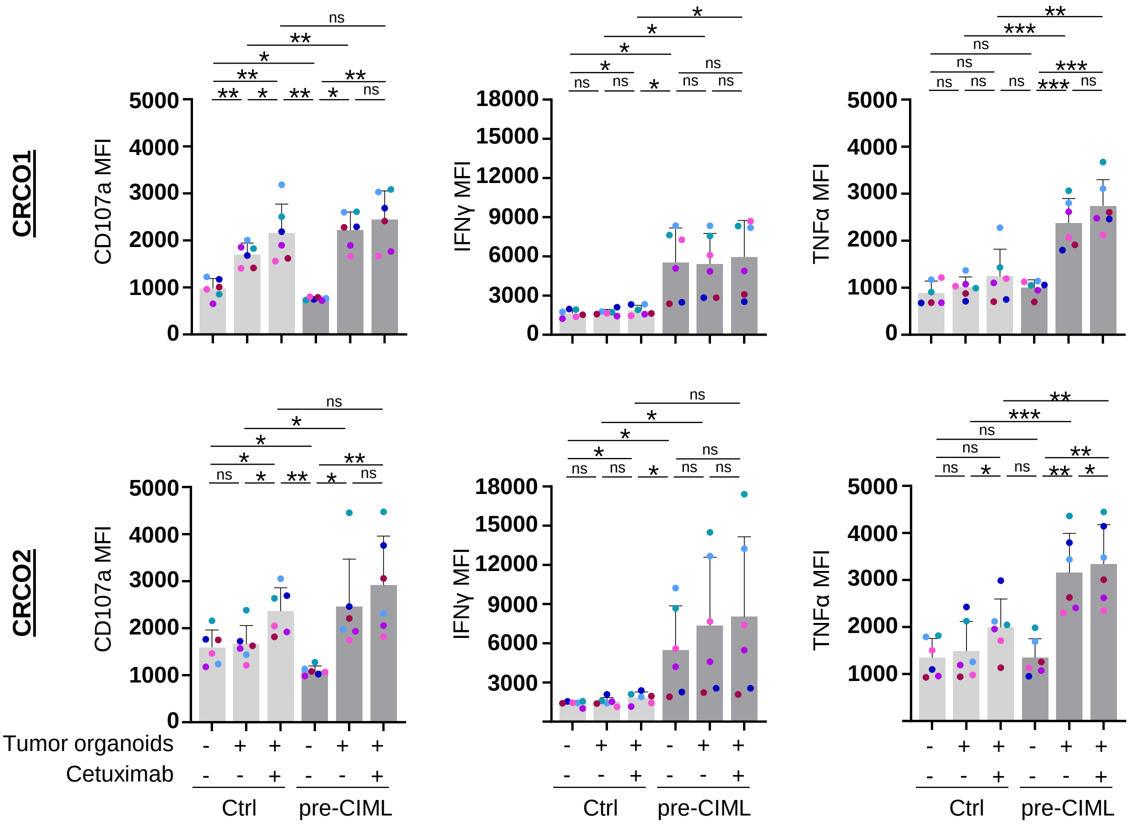

Supplemental figure 6. MFI of NK cell degranulation marker CD107a and cytokine production in response to organoids. Control or pre-CIML NK cells were co-cultured at 2:1 E:T ratio with tumor organoids (CRCO1 and CRCO2) in the presence or absence of 1 µg/mL cetuximab. Bar graphs showing MFI of CD107a, IFN-γ or TNF-α within the respective CD107a+, IFN-γ+ or TNF-α+ NK cell populations. Data are the mean ± SD of individual color-coded donors (n=6). Statistical comparisons used paired t-tests, with significance values indicated as: *($p<0.05$), **($p<0.01$), ***($p<0.001$).

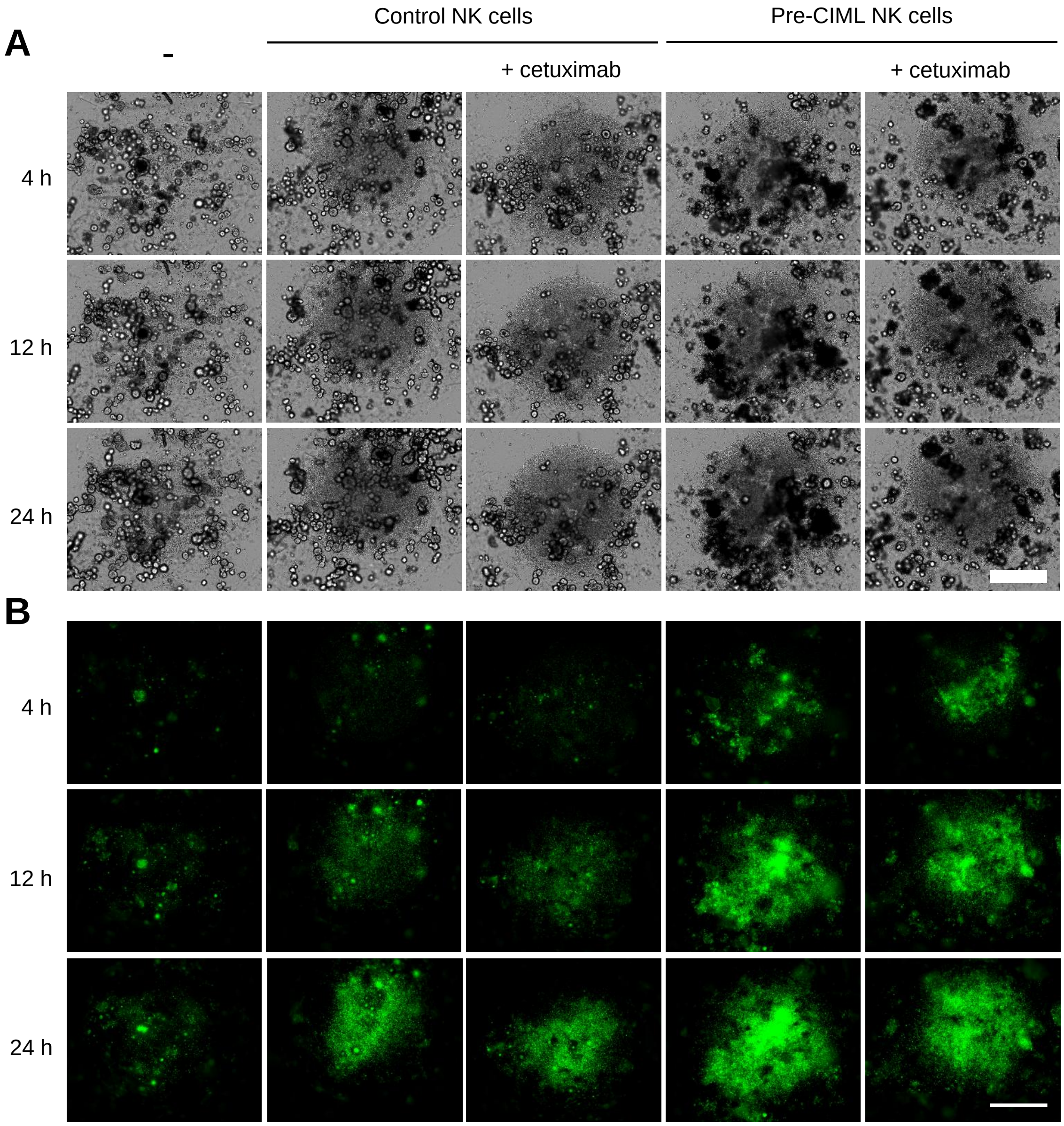

Supplemental figure 7. Representative images of NK cell-mediated cytotoxicity against patient-derived CRCO1 organoid. CRCO1 was cultured alone or co-cultured with control or pre-CIML NK cells at a 5:1 E:T ratio, in the absence or presence of 1 µg/mL cetuximab. Representative brightfield (**A**) and caspase-3/7 green fluorescence (**B**) images at 4, 12 and 24 hours of co-culture are shown to visualize caspase-dependent cell death evolution under the indicated conditions. Scale bar: 600 µm.

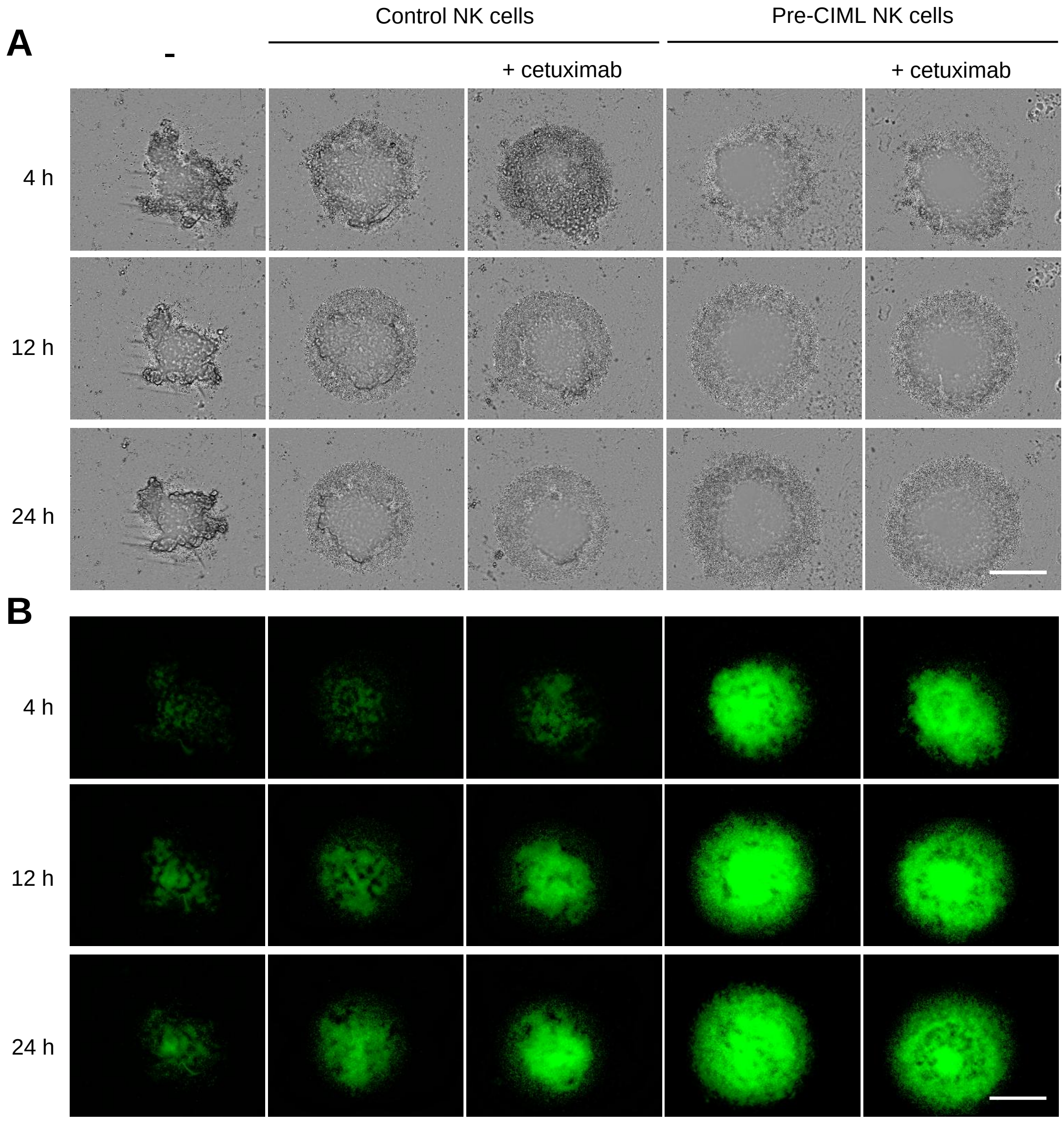

Supplemental figure 8. Representative images of NK cell-mediated cytotoxicity against patient-derived CRCO2 organoid. CRCO2 was cultured alone or co-cultured with control or pre-CIML NK cells at a 5:1 E:T ratio, in the absence or presence of 1 μg/mL cetuximab. Representative brightfield (**A**) and caspase-3/7 green fluorescence (**B**) images at 4, 12 and 24 hours of co-culture are shown to visualize caspase-dependent cell death evolution under the indicated conditions. Scale bar: 600 μm.

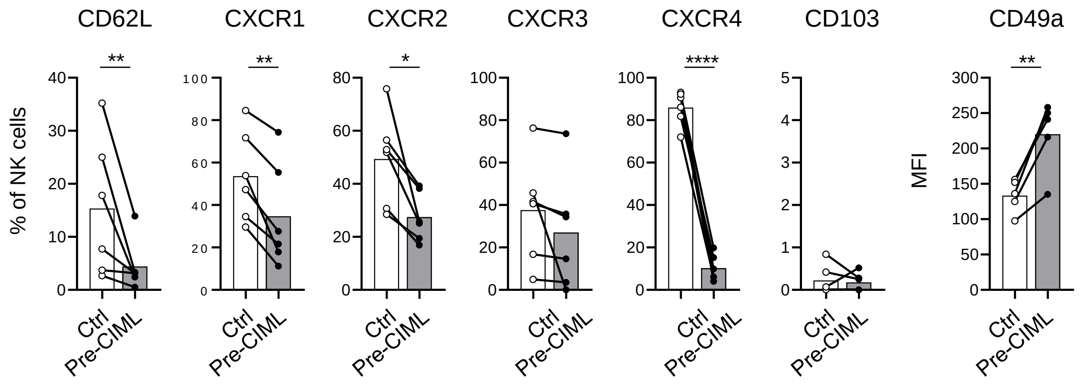

Supplemental figure 9. Expression of chemokine receptors and adhesion molecules on NK cells. Surface expression of the indicated receptors in control or pre-CIML NK cells measured by flow cytometry. The following antibodies were used: anti-CD62L-BV421 (DREG-56, BD Biosciences), anti-CXCR1-FITC (8F1, Biolegend), anti-CXCR2-PE (6C6, BD Biosciences), anti-CXCR3-PercP-Cy5.5 (1C6, BD Biosciences), anti-CXCR4-APC (12G5, BD Biosciences), anti-CD103-BV786 (Ber-ACT8, Biolegend) and anti-CD49a-PE-Cy7 (TS2/7, Biolegend). Bar graphs show the frequency of NK cells or MFI for the indicated markers, with each data point representing an independent donor. Statistical comparisons used paired t-tests, with significance values indicated as: *(p<0.05), **(p<0.01), ***(p<0.001), ****(p<0.0001).

Supplemental table 1. Mutational status of tumor models.

Tumor cells	HCT-116	A549	CRCO1	CRCO2
**KRAS**	Mutated	Mutated	Wild-type	Mutated
**PIK3CA**	Mutated	Wild-type	Mutated	Wild-type
**PTEN**	Wild-type	Wild-type	Mutated	Wild-type
**APC**	Wild-type	Wild-type	Wild-type	Mutated
**P53**	Wild-type	Mutated	Mutated	Wild-type
**MSI**	MSI-H	MSS (MMR-proficient)	MSI-L	MSI-H

Supplemental table 2. Mutational profile of tumor organoids.

	CRCO1	CRCO2
ACVR1B	Mutated	Wild-Type
ACVR2A	Mutated	Mutated
ADAMST3	Mutated	Wild-Type
AMER1	Mutated	Mutated
ARID1A	Mutated	Mutated
ASXL1	Mutated	Wild-Type
ARPC1B	Wild-Type	Mutated
ASTN2	Wild-Type	Mutated
BCL9L	Wild-Type	Mutated
CCDC168	Mutated	Mutated
CHD7	Mutated	Wild-Type
COL5A1	Mutated	Wild-Type
DIAPH1	Mutated	Wild-Type
DNAAF5	Mutated	Wild-Type
FAT1	Mutated	Wild-Type
HYDIN	Wild-Type	Mutated
IL7R	Wild-Type	Mutated
ING1	Mutated	Wild-Type
KCNB2	Mutated	Wild-Type
MGAT3	Mutated	Wild-Type
MSH3	Mutated	Wild-Type
MTUS2	Wild-Type	Mutated
MYO18B	Mutated	Wild-Type
MYO1B	Mutated	Wild-Type
NCAPD3	Mutated	Mutated
NKTR	Wild-Type	Mutated
OBSCN	Mutated	Mutated
PACSIN1	Mutated	Wild-Type
PIEZO2	Wild-Type	Mutated
PTPN12	Mutated	Mutated
RBM10	Mutated	Mutated
RBM12	Wild-Type	RBM12
SACS	Mutated	Mutated
SALL1	Wild-Type	Mutated
SOX9	Wild-Type	Mutated
SRRT	Mutated	Mutated
SYNE1	Wild-Type	Mutated
SSH1	Wild-Type	Mutated
TCF7L2	Wild-Type	Mutated
TDRD1	Mutated	Mutated
TGFBR2	Mutated	Mutated
TGFIF1	Wild-Type	Mutated
TGIF1	Wild-Type	Mutated
TP53	Mutated	Wild-Type
TRPS1	Mutated	Wild-Type
UBR1	Mutated	Wild-Type
USP5	Wild-Type	Mutated
